# Development of a nomogram integrating immune checkpoints, fibrosis indicators, and clinicopathological characteristics to predict overall survival in pancreatic cancer: a retrospective analysis

**DOI:** 10.3389/fimmu.2025.1688440

**Published:** 2025-09-22

**Authors:** Dailei Qin, Kewei Huang, Zehui Yao, Lingmin Jiang, Qi Zhu, Jianzhong Cao, Shengping Li

**Affiliations:** ^1^ Department of Pancreatobiliary Surgery, State Key Laboratory of Oncology in South China, Guangdong Provincial Clinical Research Center for Cancer, Sun Yat-sen University Cancer Center, Guangzhou, China; ^2^ Department of Clinical Laboratory Medicine, State Key Laboratory of Oncology in South China, Guangdong Provincial Clinical Research Center for Cancer,Sun Yat-sen University Cancer Center, Guangzhou, China

**Keywords:** pancreatic cancer, immune checkpoints, fibrosis indexes, extracellular volume, nomogram, overall survival

## Abstract

**Background:**

Pancreatic cancer (PC) remains a highly aggressive disease with a poor postoperative 5-year survival of around 25%, attributable to its immunosuppressive and fibrotic tumor microenvironment. Prognostic models that combine immune checkpoint markers with fibrotic features are still needed.

**Methods:**

We analyzed qualifying surgically resected PC specimens. Immunohistochemistry was used to evaluate PD-L1, CTLA-4, and α-SMA expression. Extracellular matrix volume (ECV) at the tumor center (ECVC) and peritumoral region (ECVP) was measured by three radiologists using single-energy CT. Collagen fraction (CF) was assessed via Masson’s trichrome staining. Multivariate Cox regression identified independent predictors of overall survival (OS); a prognostic nomogram was then developed.

**Results:**

Among 268 enrolled patients, divided into training (n=215) and validation (n=53) sets via Five-fold cross-validation, PD-L1 expression correlated positively with α-SMA, T stage, and N stage. Multivariate analysis identified α-SMA H-score, Masson-CF, ECVC, ECVP, T stage, N stage, CA19-9, neutrophil-to-lymphocyte ratio (NLR), vascular invasion, and chemotherapy as independent OS predictors. The nomogram integrating these factors outperformed TNM staging in predicting OS.

**Conclusion:**

High PD-L1 expression is associated with enhanced fibrosis, greater tumor burden, and nodal metastasis in PC. Patients exhibiting elevated PD-L1 levels, significant fibrotic burden, advanced T or N stage, or increased NLR demonstrate reduced OS. The developed nomogram enhances individualized prediction of OS. These findings support the hypothesis that combining immune checkpoint blockade, TGF-β inhibition, and chemotherapy may represent a promising therapeutic strategy for PC patients with high PD-L1 expression and pronounced fibrosis.

## 1 Introduction

PC is an aggressive malignancy within the gastrointestinal tract (1). PC is characterized by extensive local invasion, early systemic spread, and a notable resistance to chemotherapy and radiotherapy (2). The median overall survival for PC patients is below 6 months, with a five-year survival rate hovering around 7% (3). Recently, the combination of surgical resection with adjuvant chemotherapy presents the sole option for extended survival or potential cure in PC patients (4). Nonetheless, the 5-year survival rate for resected PC patients remains disappointingly below 20% (5). Therefore, in addition to early diagnosis and treatment for the timely detection and management of PC, the introduction of novel therapeutic agents represents one of the key strategies for improving postoperative survival in patients following curative resection (6, 7).

Over the past decade, cancer immunotherapy has transitioned from an experimental concept to a pillar of oncologic care. Immune-checkpoint blockade combined with chemotherapy has recently shown meaningful activity in PC. In a 2022–2024 single-center cohort of 57 metastatic patients, adding a PD-1 inhibitor to AG or mFOLFIRINOX significantly improved the objective response rate (42.9% vs 17.2%, P = 0.02), prolonged median progression-free survival (7.3 vs 5.8 months; HR 0.64, 95% CI 0.46–0.89), and extended overall survival (12.0 vs 10.2 months; HR 0.71, 95% CI 0.52–0.96) compared to chemotherapy alone, without increasing grade ≥3 toxicity (8). A real-world Chinese study (2020–2024) of 112 patients across five centers showed that PD-1/PD-L1 inhibitors combined with chemotherapy achieved an objective response rate of 26%, a disease control rate of 71%, and a median overall survival of 10.4 months, outperforming historical controls (9). However, the phase II trial combining durvalumab (anti-PD-L1) and tremelimumab (anti-CTLA-4) reported an ORR of only 3.1%, with no responses observed in either monotherapy arm of PC patients (10). Similarly, pembrolizumab or nivolumab used as single-agent PD-1 blockade produced no radiographic responses in unselected PC cohorts (11). Thus, for PC patients, combination therapy with immunotherapy and chemotherapy demonstrates superior efficacy compared to either immunotherapy or conventional chemotherapy alone. Nevertheless, identifying biological indicators that accurately reflect tumor immunogenicity remains essential for achieving precision medicine.

Although multiple factors including oncogenic KRAS mutations, hypoxia-induced metabolic stress, and the accumulation of myeloid-derived suppressor cells (MDSCs) and regulatory T cells (Tregs) shape the immune microenvironment of PC, two key drivers underlie its profound immunosuppression: dysregulated immune-checkpoint pathways and a rigid, desmoplastic fibrotic stroma (12–15). First, tumor cells, cancer-associated fibroblasts (CAFs), and infiltrating myeloid populations consistently express PD-L1, whereas tumor-infiltrating lymphocytes (TILs) upregulate PD-1 (16). Concurrently, CTLA-4 on regulatory T cells outcompetes CD28 for CD80/86 binding, suppressing cytotoxic T-cell responses and driving T-cell exhaustion (17). Second, the fibroblasts deposit a dense, collagen-rich extracellular matrix that mechanically traps CD8^+^ T cells in peritumoral cuffs, elevates interstitial pressure, and compresses vasculature exacerbating hypoxia (18). This physical barrier not only restricts immune cell infiltration but also transcriptionally reinforces PD-L1 expression through HIF-1α stabilization (19). Therefore, a single biomarker often fails to accurately assess tumor immunogenicity. Only multi-parameter assessment approaches can comprehensively reflect the tumor microenvironment (TME) status and thereby predict clinical outcomes. However, there is still a lack of an integrated prognostic model incorporating immune checkpoint expression profiles, fibrosis levels, and clinical parameters to predict long-term survival following curative resection in PC patients.

In this study, we first quantified immune checkpoint expression (PD-L1 and CTLA-4) in 268 pancreatic cancer patients following radical resection. Subsequently, tumor fibrotic burden was comprehensively assessed through integrated radiological-histopathological analysis. Multivariate analysis identified PD-L1 expression, fibrotic indices, NLR, T stage, N stage, and key clinical variables as independent prognostic determinants of OS. We further developed a clinically applicable nomogram to stratify OS probability. Moreover, validation studies confirmed the superior predictive performance of this integrated nomogram model compared to the TNM staging system.

## 2 Material and methods

### 2.1 The patients’ enrollment, grouping and relevant ethical approval

Patients who underwent radical resection for PC between January 2008 and December 2019 were identified from medical records. A multidisciplinary team (MDT) preoperatively assessed the safety and feasibility of radical resection for each case. Key imaging data reviewed during MDT discussions included computed tomography (CT), magnetic resonance imaging (MRI), and positron emission tomography-CT (PET-CT) findings. All procedures were performed by one of three senior surgeons with specialized expertise in PC resection. Surgical strategies were individually customized based on clinical tumor characteristics and patient preferences.

**Figure 1:**
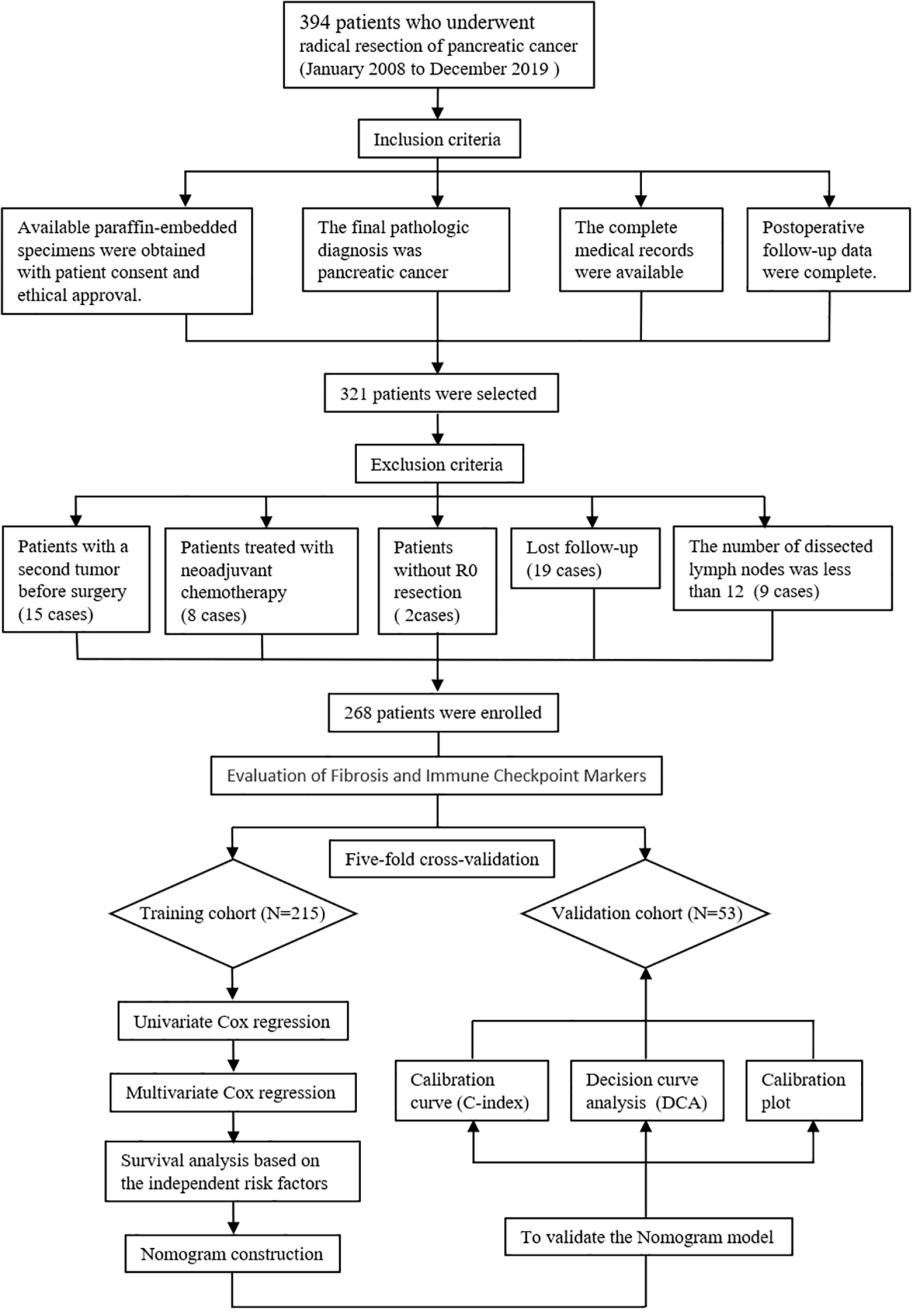
Research process diagram.

The inclusion criteria were as follows: (1) The postoperative specimen from the radical resection was pathologically confirmed as PC; (2) Written informed consent was obtained from the patient prior to specimen collection, and the study protocol was approved by the Institutional Ethics Committee; (3) Complete postoperative follow-up records, including OS and detailed documentation of adjuvant chemotherapy regimens, must be available; (4) Comprehensive pathological data such as TNM stage, lymph node harvest and positivity rate, and the presence of perineural and vascular invasion were obtained; (5) Full clinical information, including age, gender, and tumor marker test results, was available. On the contrary, the exclusion criteria were represented as follows: (1) patients with second tumor before surgery, (2) patients who received neoadjuvant chemotherapy, (3) patients without R0 resection (the margin for R0 resection was described as 1.5-2mm in the previous study) (5), (4) lost follow-up, (5) the number of dissected lymph nodes was less than 12 (20, 21). Subsequently, The cohort was partitioned through a randomized 5-fold cross-validation process. The entire dataset was first shuffled and then equally divided into 5 folds. For each validation round, one fold (approximately 20% of the data) was assigned as the validation set (n=53), while the remaining four folds (approximately 80%) were used as the training set (n=215). This process was repeated five times such that each fold served as the validation set exactly once. This strategy was employed to ensure robust model evaluation, minimize sampling bias, and maximize the use of available data for both training and validation. The detailed case inclusion process is shown in **Figure 1**.

In this study, informed consent was secured from all participants for the utilization of their medical records and pathological specimens. Additionally, the ethics committee provided in our hospital approval for the retrospective analysis conducted.

### 2.2 Immunohistochemical staining

**Figure 2:**
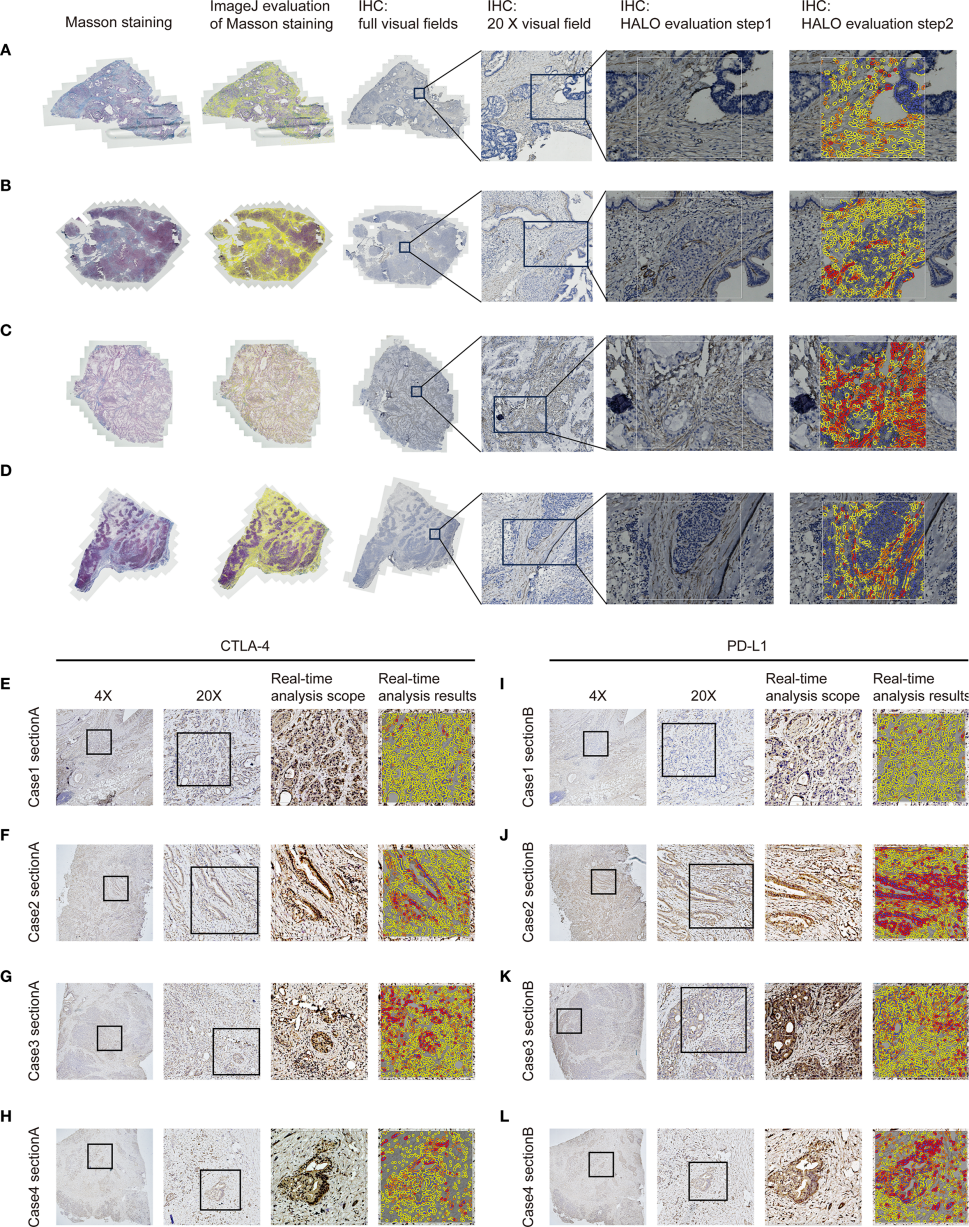
Quantification of fibrosis-related indices and immune checkpoint expression profiles in PC tissues. **(A–D)** Fibrosis extent in pancreatic cancer tissues was evaluated by IHC and Masson’s trichrome staining, with quantitative analysis of staining parameters conducted using the HALO image analysis platform and ImageJ software. **(E–H)** CTLA-4 expression in pancreatic carcinoma tissues was evaluated via immunohistochemistry with quantitative assessment performed on the HALO platform. **(I–L)** PD-L1 expression in pancreatic carcinoma tissues was evaluated via immunohistochemistry with quantitative assessment performed on the HALO platform.

Immunohistochemical staining was performed on 4-μm formalin-fixed paraffin-embedded PC tissue sections mounted on charged slides. Following deparaffinization and rehydration, heat-induced epitope retrieval was conducted in citrate buffer (pH 6.0) at 95 °C for 20 minutes. Sections were incubated overnight at 4 °C with rabbit monoclonal antibodies against α-SMA (clone ARC1912), used at a dilution of 1:200, CTLA-4 (clone ARC57390), used at a dilution of 1:200, and PD-L1 (clone ARC2478), used at a dilution of 1:200, followed by detection using HRP-polymer secondary antibodies with 3,3’-diaminobenzidine (DAB) chromogen visualization, representative IHC images are shown in **Figures 2A–L**. Counterstaining was performed with Mayer’s hematoxylin prior to dehydration and resinous mounting. Following immunohistochemical staining, whole-slide images were acquired using a high-resolution digital slide scanner (Zeiss Axio Scan.Z1) and subsequently subjected to quantitative analysis via the HALO image analysis platform (Indica Labs), with the H-score serving as the primary quantitative metric (20, 22). For the quantification of α-SMA, analysis was confined to stromal areas. In contrast, both stromal and parenchymal regions were evaluated for CTLA-4 and PD-L1, reflecting the recognized expression of these immune checkpoints not only in cancer cells but also across various cell types within the tumor microenvironment. Staining intensity was categorized as 0 (negative), 1+ (weak), 2+ (moderate), or 3+ (strong). Using automated cell segmentation, the software calculated the percentage of cells at each intensity level and derived the H-score according to the formula: H-score = (1 × %1+) + (2 × %2+) + (3 × %3+). This H-score was subsequently subjected to ROC curve analysis based on the entire cohort to determine the optimal cut-off value. It is important to note that the ROC-derived threshold, established from the overall dataset, was applied consistently to both the training and validation sets. Values above the cut-off were classified as high expression, and those below was described as low expression.

### 2.3 Masson’s trichrome staining

Sections were sequentially treated with: (1) Weigert’s iron hematoxylin for 10 minutes to stain nuclei, (2) Biebrich scarlet-acid fuchsin solution for 10 minutes to differentiate cytoplasmic and muscle fibers, (3) phosphomolybdic-phosphotungstic acid solution for 10 minutes for differential bleaching, and (4) aniline blue solution for 5 minutes to selectively stain collagen fibers. Following each staining step, sections were rinsed in distilled water with differentiation in 1% acetic acid after aniline blue application. Dehydration was accomplished through 95% and absolute ethanol (3 changes each), cleared in xylene, and mounted with resinous medium. All procedures were conducted at room temperature with precise timing controls. Quantitative analysis of stained sections was performed with ImageJ software, with collagen deposition/positive areas measured by threshold-based segmentation. The above detection and processing results are shown in **Figures 2A–D**.

### 2.4 The fibrosis assessment based on the radiology

**Figure 3:**
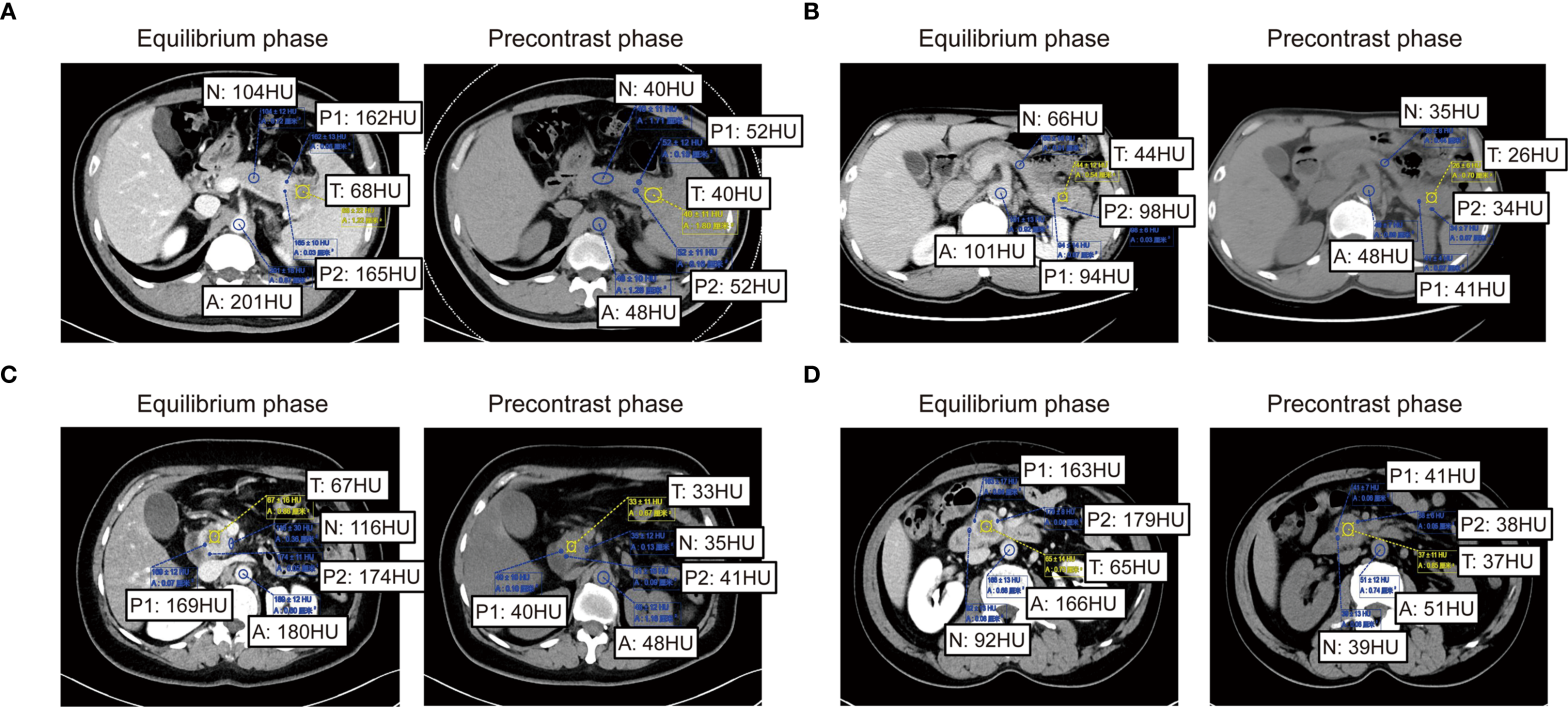
Imaging-based quantification of PC fibrosis metrics. **(A–D)** Quantitative presentation and comparison of HU values across tumor core, tumor periphery, normal pancreatic parenchyma HU values, and abdominal aorta HU values in identical patients during pre-contrast and equilibrium phases of contrast-enhanced CT.

Extracellular volume (ECV) derived from preoperative SECT was assessed through blinded analysis by three independent radiologists. Both non-contrast and contrast-enhanced CT scans were acquired under standardized imaging protocols to ensure technical consistency. To minimize motion artifacts, patients were instructed to maintain breath-hold during scanning; for those unable to comply, iterative reconstruction algorithms were employed to mitigate motion-related degradation. Key acquisition parameters included a tube voltage of 120 kV, automatic tube current modulation (range: 100–300 mA), slice thickness of 1–2 mm, reconstruction interval of 1 mm, pitch of 0.8–1.2, and a rotation time of 0.5 seconds. All images were reconstructed using a standard soft-tissue kernel. Regions of interest (ROIs) encompassed both hypodense tumor cores and relatively hyperdense tumor periphery within each patient, with the aortic lumen serving as the reference compartment. To ensure reproducible ECV quantification, a standardized protocol was applied. Three blinded readers independently measured each case, with a predefined acceptable variability of 2%. If all values agreed within this range, their mean was taken. If one value differed by >2% from two concordant readings, it was excluded and the mean of the remaining two was used. Where all three diverged without a clear outlier, a senior arbiter provided a definitive measurement or the case was re-evaluated. All procedures were documented for transparency. Clinicians solely delineated tumor and peritumoral boundaries on CT images without participating in subsequent ROI selection or ECV quantification. The ΔTumor, calculated as the difference in Hounsfield Units (HU) between the tumor center’s equilibrium phase (180 seconds post-contrast medium administration) and precontrast phase, and ΔPeritumor, as the difference in HU between the tumor periphery equilibrium and precontrast phases. ΔAorta represents the difference in HU between the aortic region’s equilibrium and precontrast phases. Subsequently, the relevant data calculations according to the following formula: ECVC = (100 - hematocrit) * ΔTumor/ΔAorta. Similarly, ECVP = (100 - hematocrit) * ΔPeritumor/ΔAorta (23–25). ECVC and ECVP were then employed in subsequent correlation analyses. The above detection and processing results are shown in **Figures 3A–D**. Thresholds for both ECVC and ECVP were determined using ROC curve analysis. Additionally, Spearman correlation analysis was performed between ECVC/ECVP values and both α-SMA H-score and Masson-CF to ensure consistency in fibrosis assessment between imaging and histopathological evaluations.

### 2.5 Collection of clinicopathological characteristics

The clinicopathological factors included in this research were chosen from the previous study focused on prognostic analyses (26–28). The pathological factors analyzed included tumor size, differentiation, lymph node metastasis, vascular invasion, lymphatic invasion, and adjacent organ involvement. Vascular invasion in pathology is defined by the presence of tumor cells within an endothelial-lined space (e.g., blood or lymphatic vessels). Diagnostic confirmation requires visible tumor cells attached to the vessel wall, floating within the lumen, or surrounded by endothelium. Artifacts such as stromal retraction must be excluded, often with the aid of special stains (e.g., CD31, D2-40, or elastic stains) to highlight endothelial structures. Its identification carries prognostic significance for metastasis risk (29). Inflammation indices such as the NLR and platelet-to-lymphocyte ratio (PLR) were also evaluated. Clinical factors incorporated in this study encompassed chemotherapy status, CA19–9 levels, CEA levels, jaundice, and diabetes. PTCD (Percutaneous Transhepatic Cholangiographic Drainage) was performed preoperatively in all jaundiced patients to reduce complication risks. CA19–9 levels were measured after PTCD but before radical surgery to minimize confounding by false elevations.

Chemotherapy regimens in this study were selected according to the National Comprehensive Cancer Network (NCCN) guidelines (2021 Version 2.0) for PC. Treatment decisions also incorporated patient preferences and Eastern Cooperative Oncology Group Performance Status (ECOG PS). For patients with better physical status (ECOG PS 0-1), the preferred regimens included FOLFIRINOX (oxaliplatin, irinotecan, leucovorin, fluorouracil), AG (nab-paclitaxel plus gemcitabine), or GS (gemcitabine plus S-1). Patients with poorer overall physical status (ECOG PS 2-5) received gemcitabine or S-1 monotherapy.

### 2.6 Follow-up

Follow-up commenced one-month post-discharge, with patients undergoing quarterly outpatient reviews. Each review routinely included abdominal and chest CT plus CA19-9, CA125, and CEA assessments. Telephonic follow-up was utilized for patients with limited outpatient access. The follow-up period extended from enrollment until loss to follow-up, mortality, or final contact.

### 2.7 Statistical analysis

Patient characteristics between training and validation groups were compared using the chi-square test. Independent prognostic factors for OS were identified through multivariable Cox regression analysis. The association between independent risk factors and OS was assessed using Kaplan-Meier methods, employing the log-rank test for non-crossing survival curves and landmark analysis for crossing curves. The correlation analysis among immune checkpoints, fibrosis indices, T stage, and N stage was performed using analysis of variance (ANOVA). The nomogram’s predictive performance was evaluated against TNM-stage models using concordance indexes (C-indexes), calibration plots, and decision curve analysis (DCA). A two-tailed P-value < 0.05 was considered statistically significant. All analyses were performed using SPSS (v22.0) and R (v4.2.2; R Development Core Team). The following R packages were utilized: getsummary, tidyverse, survival, plyr, broom, forestmodel, ggplot2, rms, survminer, and ggDCA.

## 3 Results

### 3.1 Patient’s enrollment and grouping

A cohort of 394 PC patients underwent radical surgery between January 2008 and December 2019. Following application of inclusion criteria, 321 patients remained eligible. Exclusion criteria eliminated 53 cases: Preoperative secondary malignancies (n=15), Neoadjuvant chemotherapy (n=8), Non R0 resection (n=2), Loss to follow-up (n=19), <12 dissected lymph nodes (n=9). The final study population comprised 268 PC patients, stratified into training (n=215) and validation (n=53) sets using Five-fold cross-validation. For the entire cohort, median OS was 36.9 months with a 5-year OS rate of 35.9%.

### 3.2 Quantification of immune, fibrotic relevant biomarkers

**Figure 4:**
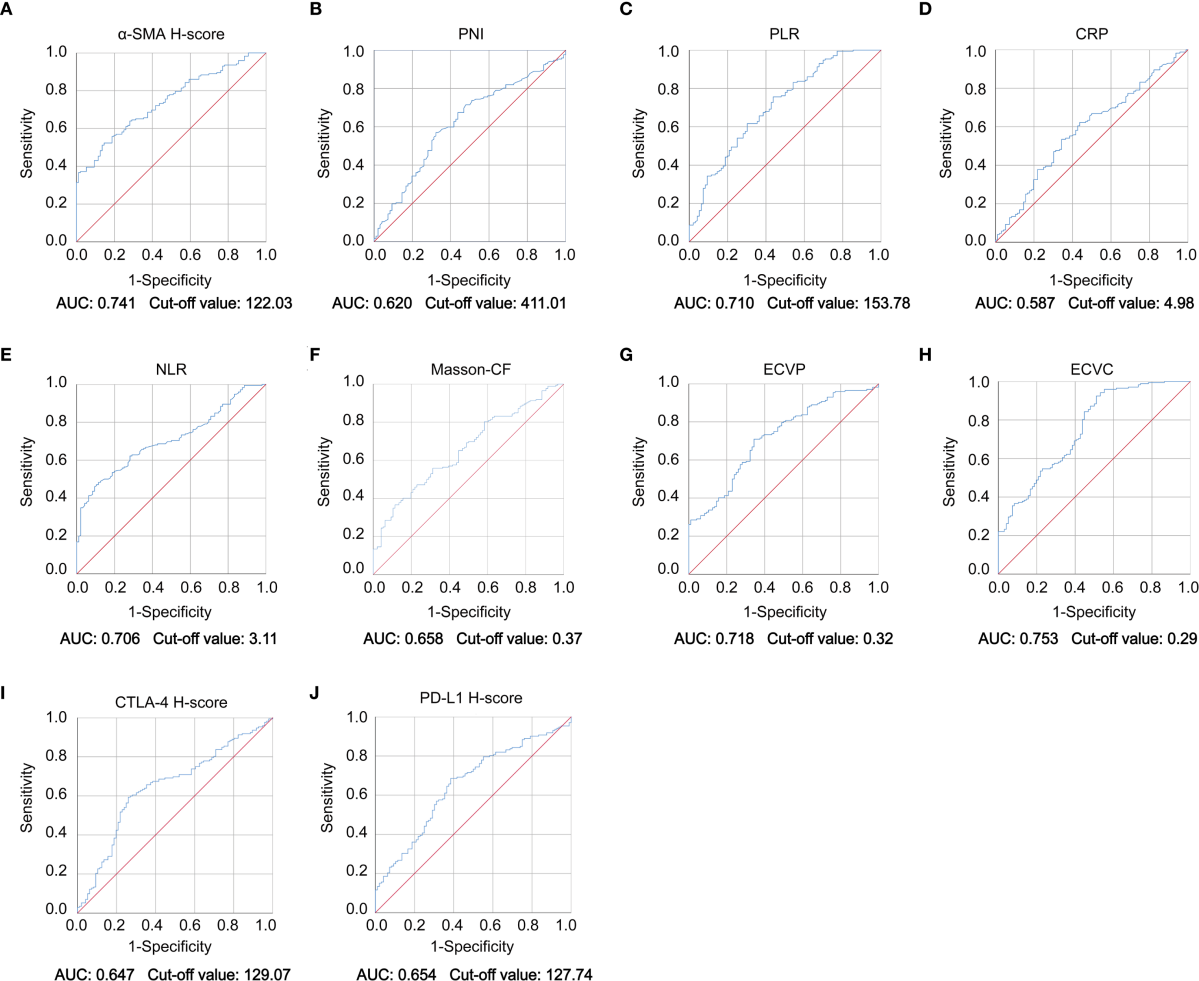
ROC curve of immune checkpoint levels, fibrotic indices, and clinicopathological parameters. **(A–J)** ROC curve analysis demonstrating diagnostic performance of α-SMA H-score, PNI, PLR, CRP, NLR, Masson-CF, ECVP, ECVC, CTLA-4 H-score, and PD-L1 H-score biomarkers with corresponding AUC values and optimal cut-off points.

**Figure 5:**
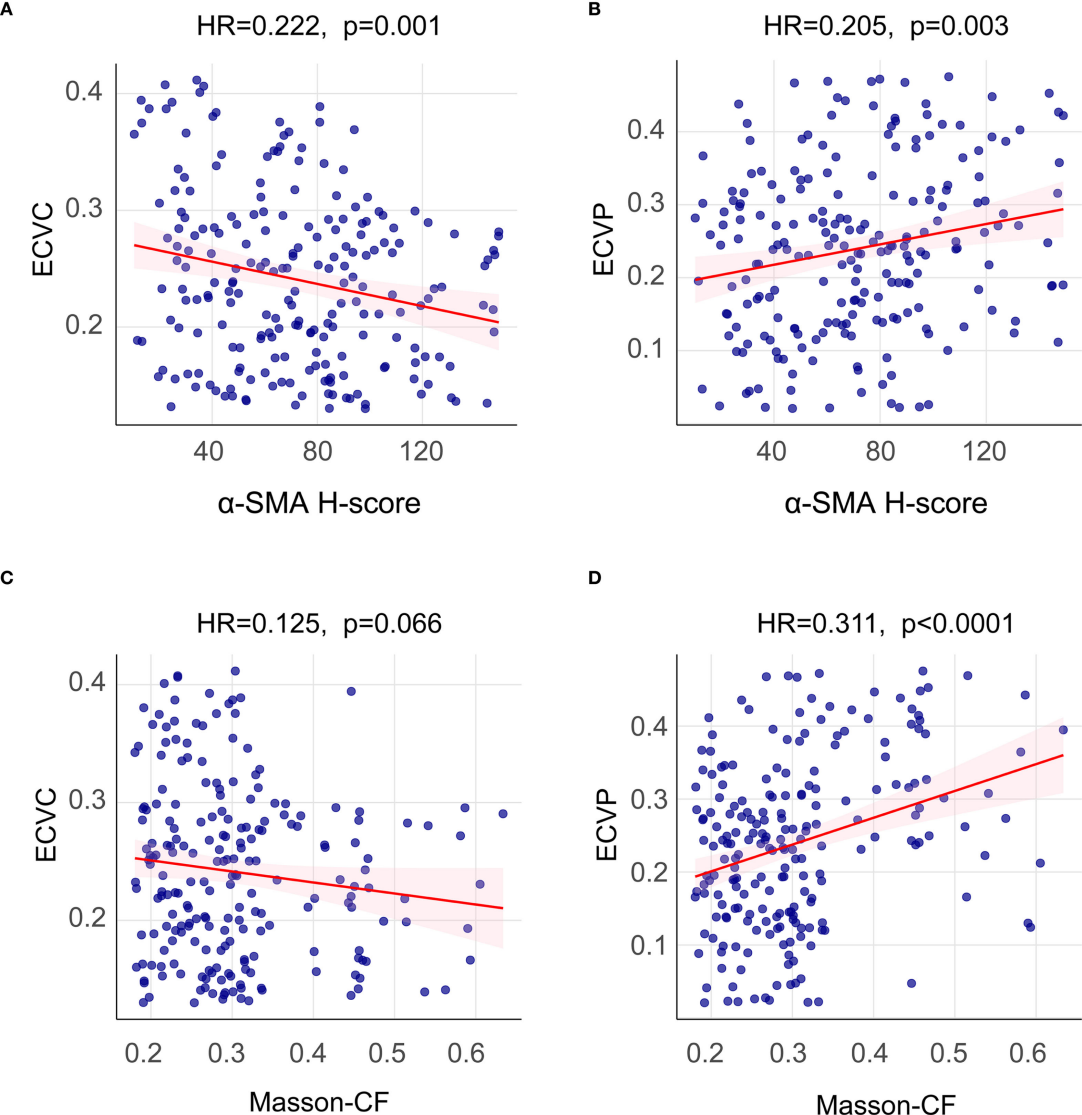
Spearman correlation analysis between imaging-based and histopathological fibrosis quantification. **(A)** Spearman correlation analysis between α-SMA H-score and ECVC. **(B)** Spearman correlation analysis between α-SMA H-score and ECVP. **(C)** Spearman correlation analysis between Masson-CF and ECVC. **(D)** Spearman correlation analysis between Masson-CF and ECVP.

This study quantified the following immune-related biomarkers: PD-L1 H-score, cytotoxic CTLA-4 H-score, NLR, PLR, PNI, and CRP. Results are expressed as mean ± standard deviation: PD-L1 = 134.12 ± 46.04, CTLA-4 = 127.21 ± 47.44, NLR = 4.56 ± 8.03, PLR = 228.48 ± 308.61, PNI = 400.07 ± 68.25, CRP = 16.94 ± 29.00. Fibrotic biomarkers included α-SMA H-score, ECVP, ECVC, and Masson-CF, with quantitative results: α-SMA = 111.71 ± 34.75, ECVP = 0.34 ± 0.11, ECVC = 0.24 ± 0.07, Masson-CF = 0.33 ± 0.11. Cut-off values for all factors were determined using ROC curve analysis based on the entire cohort. The specific ROC curves, along with their corresponding Area Under the Curve (AUC) values and cutoff values, can be found in **Figures 4A–J**. Immune/fibrosis indexes and clinical characteristics of both groups are summarized in **Table 1**. Chi-square tests revealed statistically significant differences in PD-L1, and CTLA-4 between training and validation sets, while all other metrics showed no significant inter-set variation. To evaluate the consistency between imaging-based and histopathological assessments of fibrosis severity in the same samples, we performed Spearman correlation analysis. The results indicated significant positive correlations between α-SMA and both ECVC and ECVP. Similarly, strong agreements were observed between Masson-CF and ECVC as well as ECVP. Detailed results are presented in **Figures 5A–D**.

**Table 1:** Baseline characteristics.

Characteristic	Overall, N = 268	Training, N = 215	Validation, N = 53	P-value
Gender				0.2
Female	166 (62%)	129 (60%)	37 (70%)	
Male	102 (38%)	86 (40%)	16 (30%)	
Age				0.3
<60	137 (51%)	113 (53%)	24 (45%)	
≥60	131 (49%)	102 (47%)	29 (55%)	
Site				0.5
Head	202 (75%)	164 (76%)	38 (72%)	
Body and Tail	66 (25%)	51 (24%)	15 (28%)	
T stage				0.8
T1	23 (8.6%)	20 (9.3%)	3 (5.7%)	
T2	131 (49%)	104 (48%)	27 (51%)	
T3	114 (43%)	91 (42%)	23 (43%)	
N stage				>0.9
N0	142 (53%)	114 (53%)	28 (53%)	
N1	80 (30%)	64 (30%)	16 (30%)	
N2	46 (17%)	37 (17%)	9 (17%)	
TNM stage				0.9
stage I	86 (32%)	68 (32%)	18 (34%)	
stage II	134 (50%)	109 (51%)	25 (47%)	
stage III	48 (18%)	38 (18%)	10 (19%)	
Differentiation				>0.9
Well-Moderate	132 (49%)	106 (49%)	26 (49%)	
Poor	136 (51%)	109 (51%)	27 (51%)	
Vascular invasion				0.8
Absence	171 (64%)	138 (64%)	33 (62%)	
Presence	97 (36%)	77 (36%)	20 (38%)	
Lymphatic invasion				0.8
Absence	161 (60%)	130 (60%)	31 (58%)	
Presence	107 (40%)	85 (40%)	22 (42%)	
Neurological invasion				0.084
Absence	53 (20%)	47 (22%)	6 (11%)	
Presence	215 (80%)	168 (78%)	47 (89%)	
α-SMA				0.5
<122.03	157 (59%)	124 (58%)	33 (62%)	
≥122.03	111 (41%)	91 (42%)	20 (38%)	
Masson-CF				0.2
<0.37	201 (75%)	165 (77%)	36 (68%)	
≥0.37	67 (25%)	50 (23%)	17 (32%)	
ECVP				0.7
<0.32	113 (42%)	92 (43%)	21 (40%)	
≥0.32	155 (58%)	123 (57%)	32 (60%)	
ECVC				0.14
<0.29	190 (71%)	148 (69%)	42 (79%)	
≥0.29	78 (29%)	67 (31%)	11 (21%)	
PD-L1 H-score				**<0.001**
<127.74	123 (46%)	109 (51%)	14 (26%)	
≥127.74	145 (54%)	106 (49%)	39 (74%)	
CTLA-4 H-score				**<0.002**
<129.07	141 (53%)	123 (57%)	18 (34%)	
≥129.07	127 (47%)	92 (43%)	35 (66%)	
CRP				0.8
<4.98	137 (51%)	109 (51%)	28 (53%)	
≥4.98	131 (49%)	106 (49%)	25 (47%)	
NLR				0.3
<3.11	176 (66%)	138 (64%)	38 (72%)	
≥3.11	92 (34%)	77 (36%)	15 (28%)	
PLR				0.2
<153.78	96 (36%)	81 (38%)	15 (28%)	
≥153.78	172 (64%)	134 (62%)	38 (72%)	
PNI				0.8
<411.01	125 (47%)	101 (47%)	24 (45%)	
≥411.01	143 (53%)	114 (53%)	29 (55%)	
CA19-9				0.082
<35 U/ml	65 (24%)	57 (27%)	8 (15%)	
≥35 U/ml	203 (76%)	158 (73%)	45 (85%)	
CEA				0.15
<5 ng/ml	169 (63%)	131 (61%)	38 (72%)	
≥5 ng/ml	99 (37%)	84 (39%)	15 (28%)	
CA125				0.2
<35 U/ml	222 (83%)	175 (81%)	47 (89%)	
≥35 U/ml	46 (17%)	40 (19%)	6 (11%)	
Diabetes				0.7
Absence	203 (76%)	164 (76%)	39 (74%)	
Presence	65 (24%)	51 (24%)	14 (26%)	
Jaundice				0.4
Absence	123 (46%)	96 (45%)	27 (51%)	
Presence	145 (54%)	119 (55%)	26 (49%)	
Chemotherapy				0.4
Absence	119 (44%)	93 (43%)	26 (49%)	
Presence	149 (56%)	122 (57%)	27 (51%)	

Bold text indicates statistical significance (p < 0.05).

### 3.3 Prognostic factors for OS in PC patients

**Figure 6:**
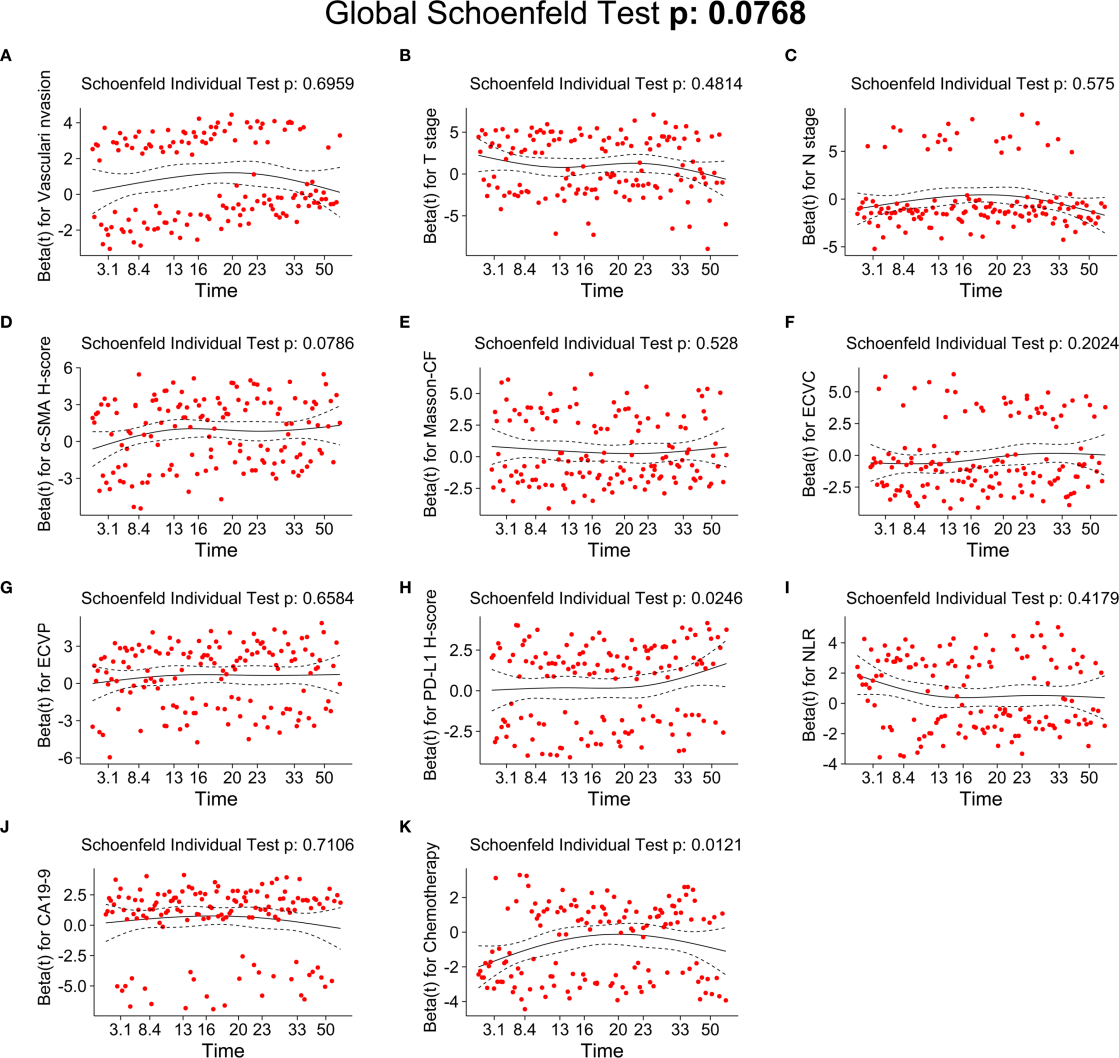
Testing of the proportional hazards assumptions for independent prognostic factors of OS. **(A)** Schoenfeld individual test of vascular invasion. **(B)** Schoenfeld individual test of T stage. **(C)** Schoenfeld individual test of N-stage. **(D)** Schoenfeld individual test of α-SMA H-score. **(E)** Schoenfeld individual test of Masson-CF. **(F)** Schoenfeld individual test of ECVC. **(G)** Schoenfeld individual test of ECVP. **(H)** Schoenfeld individual test of PD-L1 H-score. **(I)** Schoenfeld individual test of NLR. **(J)** Schoenfeld individual test of CA19-9. **(K)** Schoenfeld individual test of chemotherapy.

As detailed in **Table 2**, univariate Cox regression analysis of the training cohort assessed 26 potential prognostic factors, revealing 18 with significant association with OS in PC patients. These comprised immune factors (CRP, NLR, PLR, PNI, PD-L1, CTLA-4), fibrotic factors (α-SMA-HALO score, Masson-CF, ECVP, ECVC) and clinicopathological factors (T stage, N stage, vascular invasion, neurological invasion, CA19-9, tumor site, jaundice, and chemotherapy). Subsequently, these significant variables underwent multivariate Cox regression, ultimately identifying 11 independent OS predictors: T stage, N stage, CA19-9, NLR, vascular invasion, α-SMA-HALO score, Masson-CF, ECVP, ECVC, PD-L1 H-score, and chemotherapy administration. The detailed analysis results are presented in **Table 3**. Moreover, the raw data are presented as categorical variables in the **Supplementary Material**. To ensure the robustness of the multivariable Cox model, which incorporated a substantial number of variables, we evaluated the proportional hazards assumptions. The global test produced a p-value exceeding 0.05, demonstrating no violation of the proportional hazards assumption and thereby affirming the model’s stability. The aforementioned verification results can be found in **Figures 6A–K**.

**Table 2:** Results of univariate analysis.

Characteristics	HR	P	CI		Characteristics	HR	P	CI
Gender					CTLA-4 H-score			
Female	Reference				<129.07	Reference		
Male	0.8	0.2	0.57 - 1.12		≥129.07	2.42	**<0.001**	1.73 - 3.39
Age					PD-L1 H-score			
<60	Reference				<127.74	Reference		
≥60	1.15	0.418	0.82 - 1.6		≥127.74	2.05	**<0.001**	1.46 - 2.89
Site					Diabetes			
Head	Reference				Absence	Reference		
Body and Tail	0.51	**0.003**	0.33 - 0.8		Presence	0.91	0.648	0.61 - 1.36
Differentiation					Jaundice			
Well-Moderate	Reference				Absence	Reference		
Poor	1.19	0.296	0.86 - 1.66		Presence	2.22	**<0.001**	1.57 - 3.15
Vascular invasion					CRP			
Absence	Reference				<4.98	Reference		
Presence	1.96	**<0.001**	1.4 - 2.74		≥4.98	1.89	**<0.001**	1.35 - 2.64
Neurological invasion					NLR			
Absence	Reference				<3.11	Reference		
Presence	1.67	**0.022**	1.08 - 2.6		≥3.11	2.72	**<0.001**	1.95 - 3.8
Lymphatic invasion					PLR			
Absence	Reference				<153.78	Reference		
Presence	1.24	0.203	0.89 - 1.74		≥153.78	2.51	**<0.001**	1.72 - 3.67
T stage					PNI			
T1	Reference				<411.01	Reference		
T2	2.53	**0.02**	1.16 - 5.52		≥411.01	0.54	**<0.001**	0.39 - 0.75
T3	3.57	**0.001**	1.64 - 7.78		CA19-9			
N stage					<35 U/ml	Reference		
N0	Reference				≥35 U/ml	1.81	**0.004**	1.2 - 2.73
N1	1.28	0.206	0.87 - 1.89		CEA			
N2	2.31	**<0.001**	1.51 - 3.54		<5 ng/ml	Reference		
α-SMA H-score					≥5 ng/ml	0.94	0.741	0.67 - 1.33
<122.03	Reference				CA125			
≥122.03	2.96	**<0.001**	2.11 - 4.16		<35 U/ml	Reference		
Masson-CF					≥35 U/ml	0.87	0.534	0.55 - 1.36
<0.37	Reference				Chemotherapy			
≥0.37	2.86	**<0.001**	2 - 4.08		Absence	Reference		
ECVC					Presence	0.67	**0.017**	0.48 - 0.93
<0.29	Reference							
≥0.29	0.55	**0.002**	0.38 - 0.81					
ECVP								
<0.32	Reference							
≥0.32	2.33	**<0.001**	1.63 - 3.34	2.33				

Bold text indicates statistical significance (p < 0.05).

**Table 3:** Results of multivariate analysis.

Characteristics	HR	CI	P	Characteristics	HR	CI	P
Site				CTLA-4 H-score			
Head	Reference			<129.07	Reference		
Body and Tail	1	0.59 - 1.71	0.995	≥129.07	1.27	0.82 - 1.97	0.282
Vascular invasion				PD-L1 H-score			
Absence	Reference			<127.74	Reference		
Presence	2.15	1.48 - 3.12	**<0.001**	≥127.74	1.58	1.08 - 2.31	**0.019**
Neurological invasion				Jaundice			
Absence	Reference			Absence	Reference		
Presence	1.51	0.93 - 2.44	0.092	Presence	1.01	0.65 - 1.56	0.969
T stage				CRP			
T1	Reference			<4.98	Reference		
T2	1.75	0.76 - 4.01	0.188	≥4.98	1.1	0.75 - 1.61	0.636
T3	2.94	1.28 - 6.73	**0.011**	NLR			
N stage				<3.11	Reference		
N0	Reference			≥3.11	2.05	1.35 - 3.1	**<0.001**
N1	1.27	0.82 - 1.95	0.279	PLR			
N2	3.42	2.03 - 5.75	**<0.001**	<153.78	Reference		
α-SMA H-score				≥153.78	1.12	0.72 - 1.75	0.604
<122.03	Reference			PNI			
≥122.03	1.61	1.02 - 2.55	**0.042**	<411.01	Reference		
Masson-CF				≥411.01	0.71	0.49 - 1.04	0.08
<0.37	Reference			CA19-9			
≥0.37	1.56	1.02 - 2.4	**0.042**	<35 U/ml	Reference		
ECVC				≥35 U/ml	1.59	1.01 - 2.5	**0.046**
<0.29	Reference			Chemotherapy			
≥0.29	0.6	0.38 - 0.94	**0.027**	Absence	Reference		
ECVP				Presence	0.44	0.3 - 0.64	**<0.001**
<0.32	Reference						
≥0.32	1.75	1.14 - 2.7	**0.011**				

Bold text indicates statistical significance (p < **0.05).**

### 3.4 Survival analysis for independent prognostic factors

**Figure 7:**
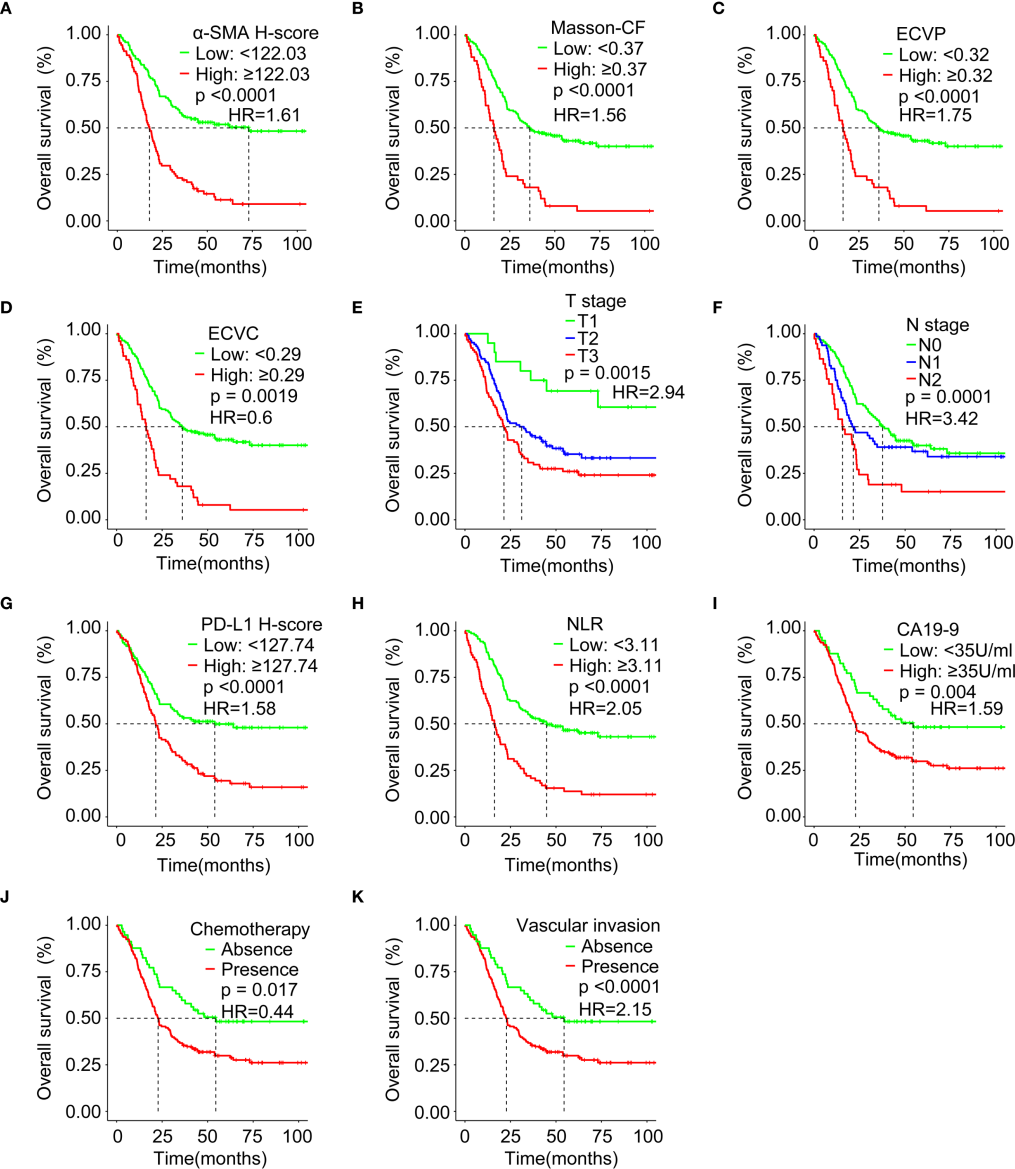
Survival analysis evaluating associations between immune checkpoints, fibrotic indices, clinicopathological factors, and OS. **(A)** Correlation between α-SMA H-score and OS in the training cohort. **(B)** Correlation between Masson-CF and OS in the training cohort. **(C)** Correlation between ECVP and OS in the training cohort. **(D)** Correlation between ECVC and OS in the training cohort. **(E)** Correlation between T Stage and OS in the training cohort. **(F)** Correlation between N Stage and OS in the training cohort. **(G)** Correlation between PD-L1 H-score and OS in the training cohort. **(H)** Correlation between NLR and OS in the training cohort. **(I)** Correlation between CA19–9 and OS in the training cohort. **(J)** Correlation between chemotherapy and OS in the training cohort. **(K)** Correlation between vascular invasion and OS in the training cohort. HR: hazard ratio.

Higher α-SMA-HALO scores, elevated ECVP values, and increased Masson-CF correlated with reduced OS, as shown in **Figures 7A–C**. Conversely, lower ECVC was associated with poorer OS, the results can be found in **Figure 7D**. Patients with T1-stage PC demonstrated significantly better OS compared to T2/T3 stages, while advanced N-stage disease showed a progressive decline in survival, as illustrated in **Figures 7E, F**. Moreover, elevated PD-L1 expression, increased NLR, and higher CA19–9 levels, all independently predicted diminished OS, as depicted in **Figures 7G–I**. Patients receiving adjuvant chemotherapy demonstrated improved survival outcomes, whereas those with vascular invasion exhibited significantly poorer OS, as illustrated in **Figures 7J, K**.

### 3.5 Correlation analysis between immune, fibrosis and clinical pathological Indicators

**Figure 8:**
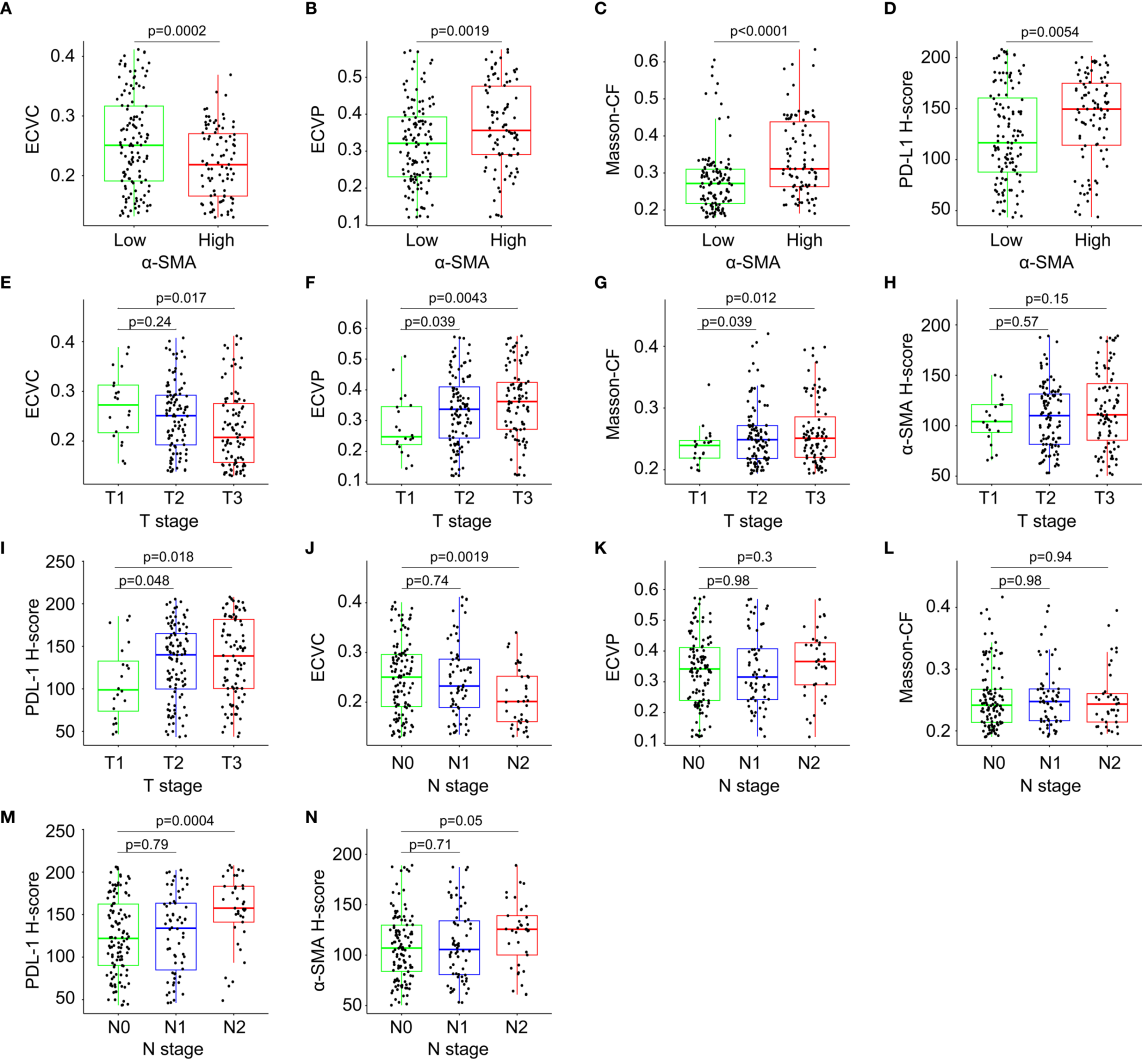
Correlation analysis among immune checkpoint expression levels, quantitative fibrotic indices, T-stage, and N-stage. **(A)** Correlation between α-SMA-HALO score and ECVC. **(B)** Correlation between α-SMA-HALO score and ECVP. **(C)** Correlation between α-SMA-HALO score and Masson-CF in the training cohort. **(D)** Correlation between α-SMA-HALO score and PD-L1-HALO score in the training cohort. **(E)** Correlation between T stage and ECVC. **(F)** Correlation between T stage and ECVP. **(G)** Correlation between T stage and Masson-CF. **(H)** Correlation between T stage and α-SMA-HALO score. **(I)** Correlation between T stage and PD-L1-HALO score. **(J)** Correlation between N stage and ECVC. **(K)** Correlation between N stage and ECVP. **(L)** Correlation between N stage and Masson-CF. **(M)** Correlation between N stage and PD-L1-HALO score. **(N)** Correlation between N stage and α-SMA-HALO score.

Patients with high α-SMA-HALO scores exhibited lower ECVC but higher ECVP values, while elevated α-SMA-HALO scores correlated with increased Masson-CF and higher PD-L1 H-scores, as demonstrated in **Figures 8A–D**. Furthermore, advancing T stage demonstrated a progressive decrease in ECVC, gradual increase in ECVP, rising Masson-CF, increased α-SMA-HALO scores, and elevated PD-L1 H-scores, **Figures 8E–I** shows these correlations. Similarly, higher N stages showed reduced ECVC values, increased ECVP, elevated Masson-CF, higher PD-L1 H-scores, and increased α-SMA-HALO scores, **Figures 8J–N** presents these findings.

### 3.6 Construction of a nomogram for OS prediction

**Figure 9:**
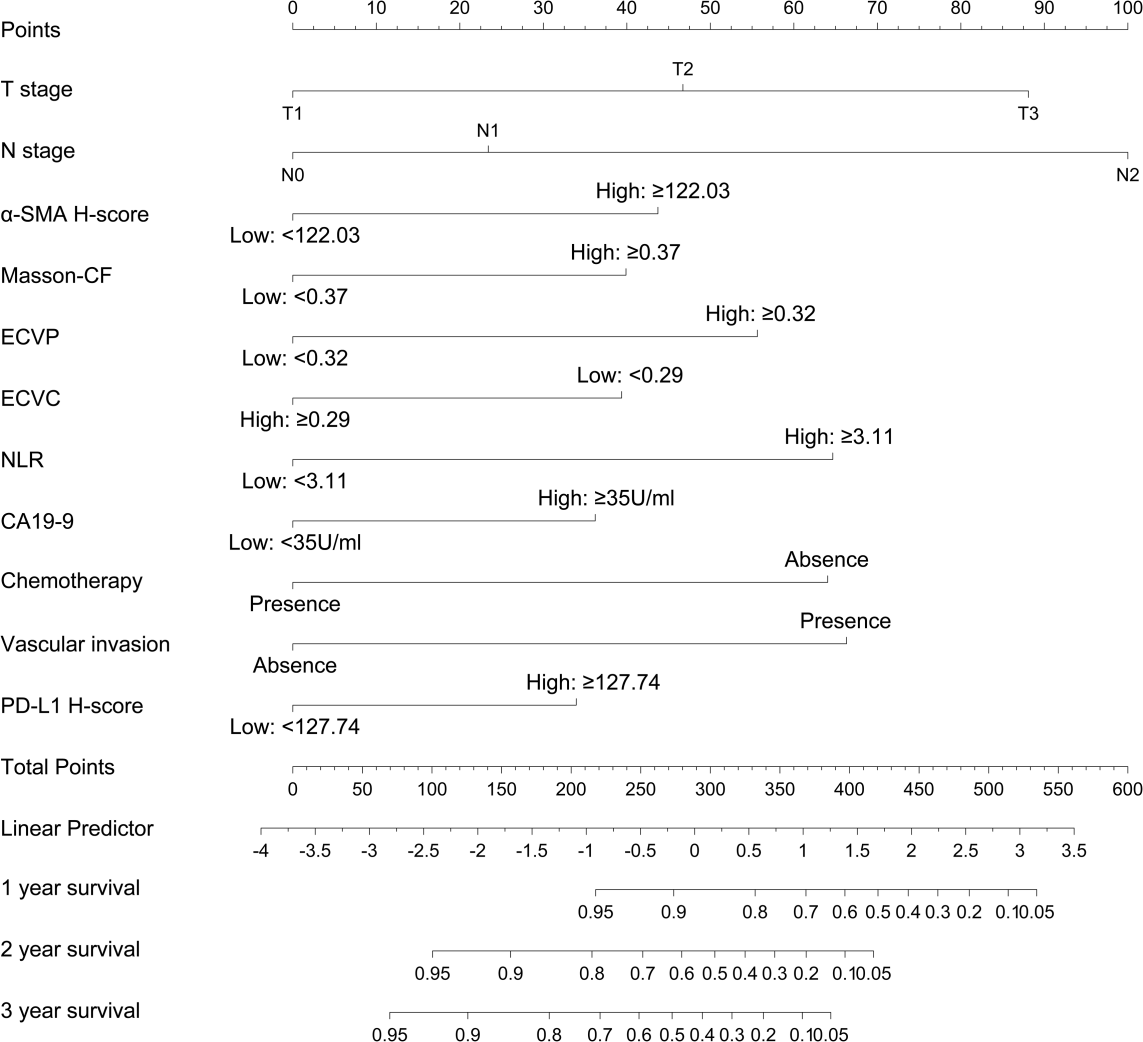
The nomogram model for OS prediction based on the independent risk factors.

A prognostic nomogram was developed to predict 1-, 2-, and 3-year OS in PC patients following radical resection, as shown in **Figure 9**. This model integrates 11 clinicopathological variables identified as independent prognostic factors through multivariate Cox regression, including T stage, N stage, α-SMA H-score, Masson-CF, ECVP, ECVC, NLR, CA19–9 level, chemotherapy administration, vascular invasion, and PD-L1 expression. Each variable contributes discrete points (range: 0-100) proportional to its prognostic weight, with N2 stage conferring maximal risk (100 points), ECVP ≥0.32 contributing 55 points, and PD-L1 H-score ≥127.74 assigned 32.5 points. The total point summation (range: 0-650) is converted to a linear predictor (range: -5 to 4) through a central axis, ultimately projecting to identically scaled probability axes (0.05-0.95) for survival estimation. For example, a linear predictor value of 0 corresponds to predicted OS probabilities of 80% at 1 year, 65% at 2 years, and 50% at 3 years.

### 3.7 Validation of constructed nomogram

**Figure 10:**
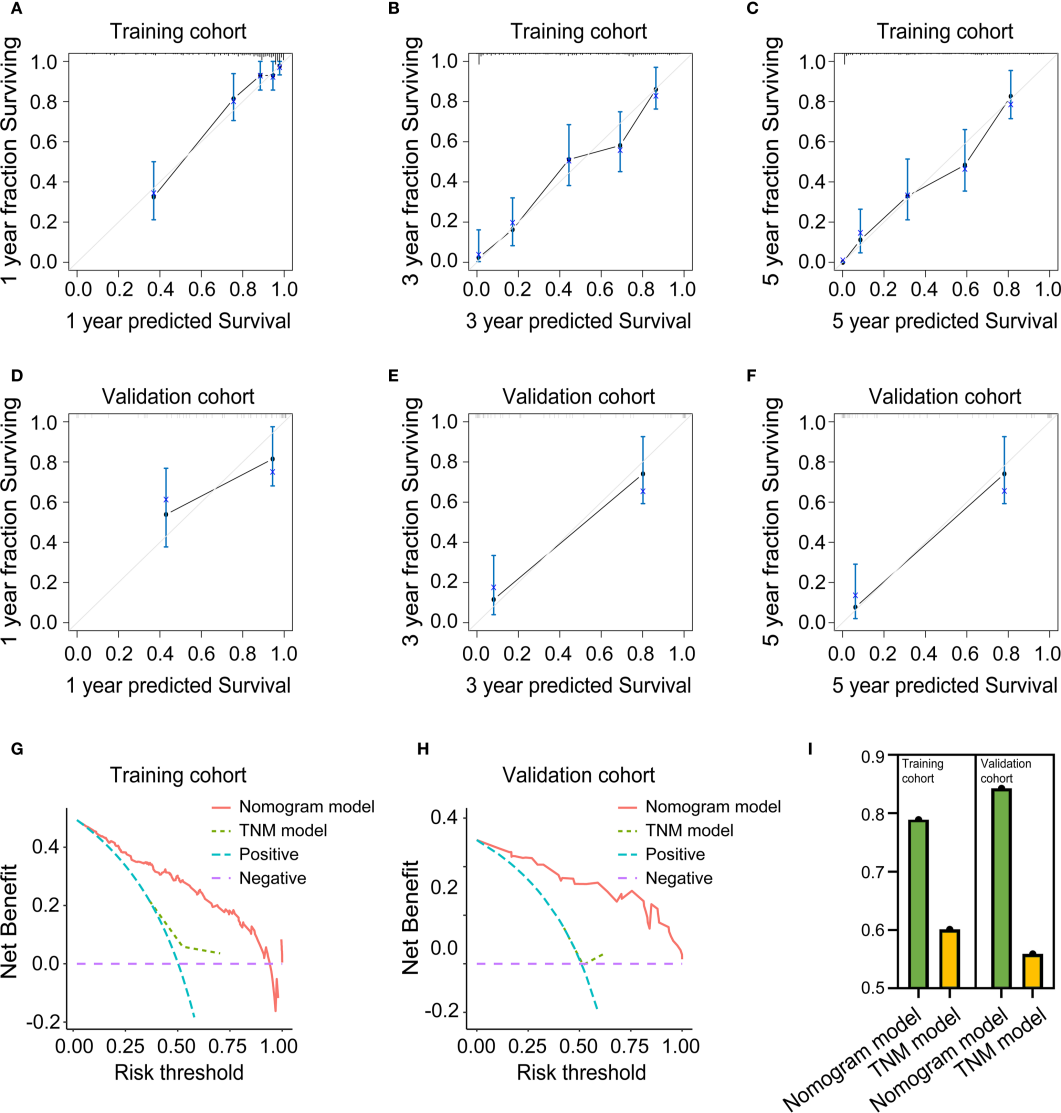
Evaluation of the predictive performance of the nomogram model. **(A)** Calibration curve for 1-year OS prediction in the training cohort. **(B)** Calibration curve for 3-year OS prediction in the training cohort. **(C)** Calibration curve for 5-year OS prediction in the training cohort. **(D)** Calibration curve for 1-year OS prediction in the validation cohort. **(E)** calibration Curve for 3-year OS prediction in the validation cohort. **(F)** calibration Curve for 5-year OS prediction in the validation cohort. **(G)** Decision curve analysis in the training cohort. **(H)** Decision curve analysis in the validation cohort. **(I)** C-Index in the training and validation cohort.

To evaluate the predictive accuracy of the nomogram, calibration plots were generated and further demonstrated close agreement between observed and predicted OS probabilities at 1, 3, and 5 years in both training and validation cohorts, with all points near the 45-degree ideal line, for details, see **Figures 10A–F**. Meanwhile, decision curve analysis demonstrated superior clinical utility of the nomogram model compared to the TNM staging system across both training and validation cohorts, indicating enhanced prognostic performance for clinical decision-making, the results are presented in **Figures 10G, H**. Ultimately, to assess discriminative performance, the C-index was calculated for both cohorts. The nomogram demonstrated significantly superior discrimination compared to the TNM staging system in training and validation cohorts, the comparative results can be found in **Figure 10I**.

## 4 Discussion

Previous studies indicate that the 5-year survival rate for PC patients is below 10% (3). Radical resection remains the only potentially curative treatment option for PC (4). However, despite adjuvant chemotherapy, approximately 85% of cases experience tumor recurrence, while the 5-year survival rate for resected PC is estimated to be merely 15%-25% (30–32). The immunological microenvironment plays a pivotal role in the progression and prognosis of pancreatic cancer (33, 34). This microenvironment is characterized by a dense stroma and a high presence of immunosuppressive cells, such as tumor-associated macrophages (TAMs), MDSCs, Tregs (15, 35, 36). These cells contribute to the immunosuppressive state by secreting factors that inhibit the activity of cytotoxic T cells and promote tumor growth and metastasis.

This immunological desert is orchestrated by the interplay of three dominant forces. First, oncogenic KRAS and chronic inflammation drive the recruitment and polarization of TAMs and MDSCs via CCL2, GM-CSF and CXCL12, while simultaneously inducing the expression of inhibitory ligands such as PD-L1, PD-L2, CD80/86 and galectin-9 on tumor and stromal cells (37, 38). Second, the PD-1/PD-L1 axis, CTLA-4 and LAG-3 act as pivotal immune checkpoints that blunt T-cell activation and sustain T-cell exhaustion (39–41). Third, an exuberant fibrotic stroma, generated chiefly by CAFs and pancreatic stellate cells, not only forms a physical barrier that impedes T-cell infiltration but also fuels immunosuppression through TGF-β–mediated signaling: TGF-β promotes extracellular-matrix deposition, induces regulatory T cells and directly suppresses cytotoxic T-cell function, whereas combined inhibition of TGF-β and PD-L1 in pre-clinical PDAC causes marked T-cell dependent tumor regression (40, 41).

Therefore, the expression status of immune checkpoints and the extent of tumor fibrosis may serve as important prognostic indicators for PC. However, there remains a lack of prognostic models that comprehensively integrate these two parameters. This study comprehensively assessed the relative contributions of immune checkpoint markers, fibrosis indices, and clinicopathological factors using multifactorial regression analysis. Results identified elevated PD-L1 and α-SMA expression levels, higher Masson-CF, increased ECVP, and reduced ECVC as independent risk factors for OS. Critically, the nomogram model developed from these risk factors demonstrated superior predictive performance relative to the conventional TNM staging system.

In PC, PD-L1 is expressed on 15–30% of tumor cells and on a substantial proportion of tumor-infiltrating immune and stromal cells; multivariate analyses of 453 resected tumors show that high PD-L1 mRNA or protein levels are independently associated with shorter disease-free survival (DFS) and OS (hazard ratio ≈ 1.5–2.0) (42, 43). CTLA-4 is chiefly expressed on intratumoral regulatory T cells and exhausted CD8^+^ T cells; elevated CTLA-4^+^ Treg frequencies correlate with higher tumor stage and independently predict poor OS in cohort studies (17, 44). In this study, we similarly observed that univariate Cox regression analysis revealed significant associations of both PD-L1 and CTLA-4 with OS in PC patients. However, multivariate Cox analysis demonstrated that only PD-L1 retained significance as an independent risk factor for OS. The correlation of PD-L1 with fibrosis and tumor burden in PC is intriguing yet controversial. PD-L1 expression is heterogeneous, and its prognostic value is still under debate. Some studies link high PD-L1 expression to poor prognosis, while others report conflicting results. The unique tumor microenvironment in PC may also indirectly affect PD-L1 upregulation. Further research is needed to clarify the mechanisms and prognostic significance of PD-L1 in PC (45, 46). In fact, the highly fibrotic tumor microenvironment in PC, characterized by sparse T-cell infiltration and dense MDSC accumulation, combined with KRAS-driven intrinsic immune evasion mechanisms, counteracts the theoretical “target enrichment” advantage conferred by PD-L1 overexpression (47). Consequently, current guidelines do not recommend PD-L1 expression as a biomarker for selecting PC patients eligible for immune checkpoint inhibitor monotherapy (48). Instead, they advocate for combination strategies (e.g., immunotherapy plus chemotherapy, anti-CD40 agonists, CXCR4 inhibitors, or TGF-β inhibitors) to overcome microenvironmental barriers, thereby potentially unlocking therapeutic benefits in PD-L1-high subgroups (40, 49).

Tumor fibrosis represents a critical microenvironmental factor in the progression of PC, modulating tumor growth, invasion, and response to therapy (50). Concurrently, extensive fibrosis in PC impedes intratumoral angiogenesis, resulting in a hypovascular state. This pathological characteristics manifest on contrast-enhanced CT as significantly reduced attenuation values in the central region of PC lesions, attributable to decreased vascular density (51). Conversely, the peripheral hyperdense rim surrounding PC lesions corresponds to desmoplastic reactions. This pathological process involves aberrant accumulation of extracellular matrix (ECM) components predominantly collagen fibers that generate a densely fibrotic stroma (52). This dense stromal reaction forms a hypervascular rim that demonstrates avid contrast enhancement on CT imaging, distinct from the hypovascular core of the tumor (53). In this study, elevated ECVP and reduced ECVC both independently predicted poorer OS in PC patients. This indicates that contrast-enhanced CT enables clinicians to preliminarily evaluate fibrosis severity and predict prognosis in PC patients. Furthermore, high α-SMA expression levels and Masson-CF were prognostic for diminished postoperative OS. Collectively, our findings lead us to hypothesize that PC patients with advanced fibrosis might benefit from adjunctive anti-fibrotic agents (e.g., TGF-β inhibitors, pirfenidone) combined with immunotherapy and adjuvant chemotherapy (54). This potential strategy warrants further exploration to determine if it can mitigate recurrence risk and improve long-term outcomes.

The T stage denotes tumor’s dimensions as determined by pathologists, signifying tumor burden. It also serves as a reference for gauging chemotherapeutic efficacy (55–57). The likelihood of drug-resistant clones within a tumor often correlates with the tumor size (58). This study further identified advanced T-stage as an independent prognostic factor for diminished OS in PC. Given that residual postoperative disease frequently drives recurrence and progression, adjuvant radiotherapy or margin-targeted irreversible electroporation (IRE, Nanoknife^®^) may reduce local recurrence rates and improve long-term survival in patients with large-volume tumors (59, 60). Furthermore, neoadjuvant chemotherapy could be recommended for PC patients with a high T stage prior to surgery. Neoadjuvant chemotherapy could prolong postoperative survival by diminishing tumor volume and invasion scope, ensuring radical resection, and minimizing residual disease (61, 62).

Previous studies have identified lymph node metastasis as a critical predictor of tumor progression (27, 63–65). Concurrently, the Japanese Pancreatic Society has highlighted that the presence of N9 and N16 lymph node metastasis is closely associated with tumor relapse and distant metastasis (66). Consistently, this research also found that a higher N stage correlate with a diminished OS in PC patients. Invasion into the lymphatic system is a predominant avenue for PC metastasis (67). Lymph node metastasis also marks the initial phase of PC metastasis and is pivotal for clinical staging, prognostic assessment, and survival in PC patients (68, 69). Hence, PC patients exhibiting lymph node metastasis may derive significant benefits from adjuvant chemotherapy, potentially enhancing OS outcomes.

We further performed correlation analysis on PDL1, SMA, T stage, and N stage to investigate the relationships among immune checkpoints, fibrosis extent, and tumor pathological parameters. The elevated α-SMA expression positively associated with PD-L1 levels. Mechanistically, α-SMA^+^ CAF-derived TGF-β induces Smad-dependent PD-L1 transcriptional upregulation in tumor cells, while CAF-secreted IL-6 activates JAK2/STAT3 signaling and CXCL12/CXCR4 axis engagement potentiates PI3K/AKT pathways, synergistically enhancing PD-L1 expression (70, 71). Moreover, fibrosis can alter the tumor microenvironment, leading to hypoxia and metabolic changes. These changes can induce epithelial-to-mesenchymal transition (EMT) in tumor cells, which is associated with increased PD-L1 expression (72). CAFs express fibroblast activation protein (FAP), and targeting CXCL12 from FAP-expressing CAFs has been shown to synergize with anti-PD-L1 immunotherapy, indicating that CAFs can influence PD-L1 expression and immune evasion (73). Further analysis revealed a positive correlation between α-SMA expression levels and both tumor T and N stage. This correlation may be attributable to CAF-derived TGF-β activating Smad2/3 signaling in tumor cells, which promotes EMT through E-cadherin suppression and vimentin/MMP-2/9 upregulation, consequently enhancing cellular motility and intravasation potential (73–75). Meanwhile, CAF-derived VEGF-C, CXCL12 and IL-6 induce peritumoral lymphangiogenesis through VEGFR-3 on lymphatic endothelial cells, while hypoxia-activated tumor cells secrete additional VEGF-C, creating a self-reinforcing loop that facilitates entry into draining lymph nodes (76). Moreover, elevated PD-L1 expression positively correlates with advanced T/N-stage. This association may occur through PD-L1/PD-1 binding, which suppresses cytotoxic T-cell activity and enables cancer cells to acquire stemness properties and undergo EMT (77). These PD-L1-high stem-like cells subsequently upregulate CXCR4 and VEGF-C, driving chemotaxis toward CXCL12-rich lymphatic niches and promoting peritumoral lymphangiogenesis (78, 79).

Vascular invasion profoundly impacts PC prognosis as a key determinant of disease progression and survival outcomes. Patients exhibiting vascular invasion demonstrate significantly increased risk of metastatic dissemination, contributing to the characteristically poor five-year survival rate of approximately 7% (80). Vascular invasion facilitates PC metastasis through tumor cell-endothelial interactions that compromise vascular integrity, enabling tumor cell intravasation (80). Our study demonstrated a significant correlation between vascular invasion and reduced OS in PC. Given its association with increased recurrence and metastatic risk, patients with vascular invasion should receive adjuvant chemotherapy promptly post-resection. This approach mitigates adverse prognostic effects and optimizes therapeutic outcomes in this high-risk cohort.

CA19-9, an established biomarker overexpressed in PC and other malignancies, is clinically utilized to monitor disease progression and treatment response in PC patients (81). However, its non-specificity to PC, with potential elevations in benign pancreatic conditions, hepatic diseases, and gastrointestinal disorders (82). This study identified a significant correlation between elevated CA19–9 levels and reduced OS in PC. In jaundiced patients, preoperative PTCD mitigates confounding inflammatory effects on CA19-9, enabling more accurate prognostic assessment. For patients with preoperative CA19–9 elevation, vigilant metastasis surveillance is warranted, with neoadjuvant chemotherapy considered for borderline resectable or high-risk cases.

The NLR, calculated from absolute neutrophil and lymphocyte counts in routine complete blood counts (CBC), serves as a validated biomarker of systemic inflammation and host immune status (83). Elevated NLR has been consistently linked to adverse prognosis, potentially due to its role in promoting angiogenesis, enhancing tumor cell proliferation, and increasing the risk of metastasis (84). Critically, elevated NLR reflects an immunosuppressive state that facilitates tumor progression and confers therapy resistance (83). Our study demonstrated a significant correlation between elevated NLR and reduced OS in PC patient. These patients require vigilant postoperative surveillance for tumor recurrence (85). Conversely, patients with low NLR may present a more favorable immune profile, potentially responding better to immunotherapeutic interventions (86–88).

Since the high invasive capacity of PC, micro-metastatic lesions and residual tumor foci commonly co-existed in the same patient (89–91), adjuvant chemotherapy was imminent after radical resection (92–95). The preceding research declared that chemotherapy inhibited tumor progression and metastasis (96–99). This study also demonstrated that PC patients achieving longer OS benefit from postoperative chemotherapy.

This nomogram model offers significant clinical utility by facilitating non-invasive assessment of tumor fibrosis through imaging interpretation, thereby reducing the reliance on invasive procedures such as biopsy. Meanwhile, by integrating immune checkpoint markers, fibrosis extent, and clinicopathological parameters, the model also improves the accuracy of postoperative overall survival prediction in PC, providing valuable support for clinical decision-making.

This study has several limitations. First, its single-center retrospective cohort design may constrain external validity due to regional practice variations. Multicenter controlled studies are required to validate the nomogram’s predictive capacity. The cases in this study did not receive postoperative immunotherapy. Future studies in PC patients undergoing immunotherapy, with stratified analyses, would enable more detailed exploration of the correlations among immune checkpoint expression, fibrosis extent, and immunotherapy response within the tumor microenvironment. Selection bias represents a significant concern: patients with comorbid stroke or coronary artery disease were less likely to undergo resection given elevated surgical risk compared with healthier candidates, potentially limiting the model’s applicability in high-risk populations. Although our study identified a positive correlation between α-SMA and PD-L1 expression levels through correlation analysis, these findings still require further validation via cellular experiments and possibly animal models. These additional investigations will be conducted and verified in future research. The absence of nutrition-related indicators in our final model constitutes a limitation that may restrict its predictive performance. To address this gap, subsequent studies will integrate a broader spectrum of nutritional indices. Finally, insufficient characterization of certain variables including specific chemotherapy regimens and N-stage subgroups may reduce predictive precision; incorporating these parameters could enhance the nomogram’s accuracy.

## 5 Conclusion

A significant positive correlation was observed between PD-L1 expression and both α-SMA levels and advanced T/N stage. A prognostic nomogram that incorporates α-SMA H-score, Masson-CF, ECVC, ECVP, T-stage, N-stage, CA19-9, NLR, vascular invasion, and chemotherapy status was developed and effectively predicts OS in PC. Building upon these correlations, the preliminary findings raise the possibility that combining immune checkpoint blockade, TGF-β inhibition, and chemotherapy may represent a promising therapeutic strategy for PC patients exhibiting high PD-L1 expression and stromal fibrosis.

## Data Availability

The original contributions presented in the study are included in the article/**Supplementary Material**. Further inquiries can be directed to the corresponding authors.
